# Impact of Lifestyles (Diet and Exercise) on Vascular Health: Oxidative Stress and Endothelial Function

**DOI:** 10.1155/2020/1496462

**Published:** 2020-09-26

**Authors:** Andy W. C. Man, Huige Li, Ning Xia

**Affiliations:** Department of Pharmacology, Johannes Gutenberg University Medical Center, Mainz, Germany

## Abstract

Healthy lifestyle and diet are associated with significant reduction in risk of obesity, type 2 diabetes, and cardiovascular diseases. Oxidative stress and the imbalance between prooxidants and antioxidants are linked to cardiovascular and metabolic diseases. Changes in antioxidant capacity of the body may lead to oxidative stress and vascular dysfunction. Diet is an important source of antioxidants, while exercise offers many health benefits as well. Recent findings have evidenced that diet and physical factors are correlated to oxidative stress. Diet and physical factors have debatable roles in modulating oxidative stress and effects on the endothelium. Since endothelium and oxidative stress play critical roles in cardiovascular and metabolic diseases, dietary and physical factors could have significant implications on prevention of the diseases. This review is aimed at summarizing the current knowledge on the impact of diet manipulation and physical factors on endothelium and oxidative stress, focusing on cardiovascular and metabolic diseases. We discuss the friend-and-foe role of dietary modification (including different diet styles, calorie restriction, and nutrient supplementation) on endothelium and oxidative stress, as well as the potential benefits and concerns of physical activity and exercise on endothelium and oxidative stress. A fine balance between oxidative stress and antioxidants is important for normal functions in the cells and interfering with this balance may lead to unfavorable effects. Further studies are needed to identify the best diet composition and exercise intensity.

## 1. Introduction

Obesity has become an epidemic and represents the major risk factor for several chronic diseases, including diabetes, cardiovascular diseases, and cancer [1]. Dietary modifications and physical exercise are popular among individuals who want to prevent overweight and keep fit. However, some recent studies have also suggested that the enthusiasm for the potential benefits of specific diets may exceed the current evidence supporting their implications [2, 3]. Therefore, it is very important to reappraise the risks and benefits of different diets to avoid unnecessary side effects.

The imbalance between prooxidants and antioxidants is linked to cardiovascular and metabolic diseases [4]. In normal conditions, homeostatic reactive oxygen species (ROS) act as secondary messengers in various intracellular signaling pathways in the cardiovascular system [5]. However, cellular oxidative stress is developed when the production of ROS and other oxidants exceeds the antioxidant defense [6]. Oxidative stress may lead to the subsequence oxidative modification or damage lipids, proteins, and DNA with deleterious consequences for metabolic and cardiovascular diseases [5]. Indeed, it has been shown that dietary and physical factors play an important role in modulation oxidative stress and endothelial function. Diet is a very important source of antioxidants, while exercising offers many health benefits, especially to cardiovascular system and muscle. Recent studies and media have suggested some specific diets to prevent overweight and improve cardiovascular health, including Mediterranean diet, ketogenic diet, and calorie restrictions [7–9]. However, different diets and physical factors have debatable roles in modulating oxidative stress and effects on the vascular system. The knowledge about the role of the behaviors and factors which are protective or harmful to the endothelium is still growing, and the newest information is recently summarized [10]. Since the endothelium and oxidative stress play critical roles in cardiovascular and metabolic diseases, appropriate choice of dietary and physical factors could have significant implications in the prevention of cardiovascular and metabolic diseases.

In this review, we summarize current knowledge on the impact of diet modification (including different diet styles, calorie restriction, and nutrient supplementation) and physical factors on endothelium and oxidative stress. Besides, we further discuss the friend-and-foe roles of dietary on endothelium and oxidative stress, focusing on cardiovascular and metabolic diseases.

## 2. Endothelium

Endothelium is a single layer of flat, polygonal endothelial cells that rest on the inner walls of blood vessels. Endothelium plays an important role in modulating vascular function by sensing the shear or frictional force between blood flow and vascular endothelium. Upon stimuli, such as blood flow and receptor-mediated stimulants, endothelial cells release important vasoactive substances including both vasodilating [such as endothelium-derived hyperpolarizing factors (EDHFs), prostacyclin (PGI_2_), and nitric oxide (NO)] and vasoconstricting factors [such as endothelin-1 (ET-1), thromboxane A2 (TXA2), and angiotensin II (Ang II)] to regulate vascular tone and architectures [11–13]. The activity of endothelial-derived NO or endothelium-derived relaxing factor (EDRF) plays an important role in the regulation of vascular function, blood pressure, and blood flow and has been widely used as a clinical marker of endothelial function [14, 15]. Mechanical forces elicited by the blood flow (shear stress) and pressure (cyclic strain) stimulate the gene expressions in endothelial cells and activate endothelial nitric oxide synthase (eNOS), which produces NO to regulate vascular function [16, 17]. In addition, it is known that laminar shear stress can also regulate antioxidant enzymes [18].

Vascular endothelium is the primary site of dysfunction in metabolic and cardiovascular diseases. Moreover, endothelial dysfunction is a hallmark of vascular aging [19]. Risk factors including hypertension, hypercholesterolemia, diabetes, and smoking are all associated with endothelial dysfunction [20]. Endothelial dysfunction is mainly characterized by the impairment in endothelium-dependent relaxation of blood vessels and the induction of a proinflammatory or prothrombotic state [16]. While NO inhibits platelet aggregation, smooth muscle cell proliferation, and the adhesion of monocytes to endothelial cells, depletion of NO leads to endothelial dysfunction and abnormal vascular remodeling [21]. Apart from pathological conditions, the anticontractile ability of endothelium is also significantly reduced during aging [19, 22], partly due to the decreased eNOS expression, NO bioavailability, or the soluble guanylyl cyclase (sGC) presence in the endothelium of aged arteries [23, 24]. Several pharmacological strategies including statins, angiotensin II receptor antagonists, and angiotensin-converting enzyme (ACE) inhibitors have been demonstrated to improve endothelial function in different studies [25]. Nonpharmacological interventions, such as physical activity and nutritional factors also play an important role in maintaining normal endothelial function [10].

## 3. Oxidative Stress in the Endothelium

**Figure 1:**
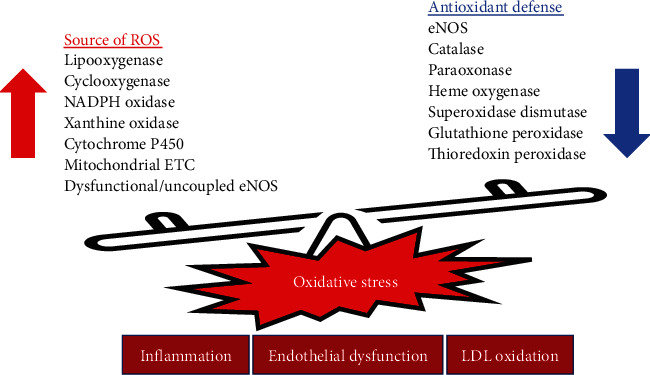
Oxidative stress occurs when the ROS production exceeds antioxidant defense. Generation of ROS is a normal physiological process. Under normal conditions, deleterious ROS are mostly removed by cellular antioxidant systems including functional endothelial nitric oxide synthase (eNOS), superoxidase dismutase, catalase, glutathione peroxidase, heme oxygenase, thioredoxin peroxidase, and paraoxonase. Sources of ROS including nicotinamide adenine dinucleotide phosphate (NADPH) oxidase, cyclooxygenase, xanthine oxidase, lipooxygenase, cytochrome P450, and dysfunctional eNOS are augmented resulting in oxidative stress and related endothelial dysfunction.

Oxidative stress occurs when the cellular production of oxidant molecules, such as ROS, exceeds antioxidants' ability to defeat these insults. Generation of ROS is a normal physiological process in aerobic organisms. Vascular ROS are important intracellular signaling messengers that regulate vascular contractility, cell growth, and vascular remodeling [26]; however, oxidative stress can trigger the pathogenesis of related cardiovascular diseases [27]. Under normal conditions, deleterious ROS are mostly removed by cellular antioxidant systems (Figure 1). Excessive ROS are known to cause lipid peroxidation and oxidative modifications of proteins and nucleic acids that cause endothelial dysfunction [28].

Oxidative stress and the associated oxidative damage are mediators of vascular injury and inflammation in many cardiovascular diseases, especially when complicated with hypertension, hyperlipidemia, and diabetes [29, 30]. The major source of oxidative stress in the arterial wall is nicotinamide adenine dinucleotide phosphate (NADPH) oxidase (NOX) [31], which is implicated in the generation of ROS and the scavenging of NO [32]. In endothelium, increased generation of ROS reduces NO bioavailability, resulting in promotion of vasoconstriction and endothelial dysfunction. Other sources of ROS in the vascular wall include mitochondrial respiratory chain and other enzymatic reactions such as cyclooxygenase (COX), xanthine oxidase (XO), lipooxygenase (LOX), cytochrome P450, and dysfunctional eNOS [33–35]. On the other hand, vascular wall contains various enzymes that can reduce the ROS burden and act as antioxidant defense systems. These include superoxidase dismutase (SOD), catalase, glutathione peroxidase (GPx), heme oxygenase (HO), thioredoxin peroxidase (TPX), and paraoxonase (PON) [33, 36, 37]. Oxidative stress can lead to the oxidation of low-density lipoprotein (LDL), which inhibits the release of EDRF more than native or unoxidized LDL [14]. Moreover, oxidized LDL (ox-LDL) is cytotoxic to endothelial cells and chemotactic for monocytes, leading to accumulation of inflammatory cells and ROS in vasculature [38, 39]. Since vascular oxidative stress is the main pathophysiological mechanism leading to blunt NO bioavailability and endothelial dysfunction, attentions to potential treatment or prevention by dietary antioxidant substances have been drawn.

## 4. Diet Effect on Endothelial Function and Oxidative Stress

Healthy lifestyle and diet are associated with significant reduction in risk of obesity, type 2 diabetes, and cardiovascular diseases [40–42]. Due to the important role of ROS in CVD as mentioned above, there has been enormous interest in the application of naturally occurring antioxidants and diet interventions to ease or prevent metabolic and cardiovascular diseases.

It has been demonstrated that dietary factors may induce significant changes on vascular reactivity [15, 43–45]. Epidemiological evidence supports the concept that diets rich in fruits and vegetables promote health and prevent the development of cardiovascular diseases [46, 47]. A variety of nutrients have shown to improve endothelial function and prevent cardiovascular diseases. Recently, attention has been focused on dietary patterns in populations with lower prevalence of cardiovascular disease, for example, Mediterranean diet. A longitudinal investigation on human subjects suggests that a healthy diet containing more lean fish, raw vegetables, and fewer high-fat dairy products is associated with less endothelial dysfunction [48]. In addition, consumption of plant-derived foods which contain micronutrients, such as fiber, antioxidant, and phytochemicals, may inhibit intracellular inflammatory signaling pathways and reduce oxidative stress [49]. Here, we summarize the recent results from the animal studies and randomized trials on diet interventions and supplements that modulate vascular functions and discuss on both their beneficial and side effects.

### 4.1. High-Fat Diet

According to American Heart Association (AHA) guidelines, diet with more than 35% of total calories from fat is regarded as high-fat diet (HFD) [50]. HFD is a common experimental diet model used to induce obesity in animals. In general, there is no doubt that HFD is associated with an increased risk of cardiovascular diseases. Early researches, working on HFD and cardiovascular risk, focused on metabolic and lipid profile abnormalities [51], while more recent studies have indicated a potential and direct effect of endothelial dysfunction induced by dietary fat intake [52, 53]. HFD is considered as a risk factor for cardiovascular disease and causes endothelial dysfunction mostly due to its association with obesity and insulin resistance [54, 55]. Indeed, single high-fat meal can already impair endothelial function transiently, in terms of flow-dependent vasoactivity in normocholesterolemic volunteers [56]. Two consecutive fat-rich meals can impair endothelial function and elevate oxidative stress markers in healthy man [57]. Four-day HFD intake can induce endothelium-dependent vasodilator dysfunction associated with diminished NO bioavailability in healthy adults [53]. These suggest that HFD could have a direct negative effect on endothelium. Nevertheless, HFD can impair endothelial function in mice and reduce the local antioxidant defense in aorta [58]. HFD may induce endothelial dysfunction, at least partly, due to triacylglycerols that reduce NO bioavailability and increase oxidative stress [52, 57].

Indeed, not all HFDs have negative effects on endothelial function, but rather depend on different types of fat [59]. High intake of saturated fat increases the risk of cardiovascular diseases and decreases endothelial fibrinolytic capacity [60]. Diet high in saturated fat has been shown to induce cholesterol-independent endothelial dysfunction and increase markers of oxidative stress in rat [61]. The habitual consumption of diet high in saturated fat is strongly associated with impaired endothelial function (reactive hyperaemia index) in young overweight adults [62]. On the other hand, diets enriched in unsaturated fatty acids seem to show beneficial effects on endothelial function [63]. Obesity induced by high unsaturated-fat diet in rat has improved vascular reactivity to leptin and does not generate endothelial dysfunction, possibly due to the increase of vascular sensitivity to leptin and leptin-induced NO bioavailability [64]. In addition, high intake of trans fatty acids can adversely affect endothelial function and increase plasma inflammatory marker including C-reactive protein (CRP), interleukin-6 (IL-6), and soluble cell adhesion molecules (sICAM-1 and sVCAM-1) according to a cross-sectional study of 730 women [65]. High trans-fat diet has also been shown to reduce endothelial function (flow-mediated dilation) [66, 67] as well as endothelial cell activation [65]. Trans-fat may cause endothelial dysfunction, at least partially, by increasing NF-*κ*B activation and impair insulin-mediated NO production in endothelial cells [68].

While HFD is relatively an experimental diet, cafeteria diet (CAF) containing a variety of highly palatable, high-salt, high-fat, and low-fiber energy dense foods, which is accessible in Western societies, is more accurately reflecting an obese diet [69]. CAF is a robust model of human metabolic syndrome with liver and adipose inflammation [70]. Indeed, both HFD and CAF can induce obesity, glucose intolerance, and insulin resistance to a comparable extent [71]. CAF can induce endothelial dysfunction in the absence of insulin resistance in rats [72], as well as vascular contractile dysfunction associated with increased oxidative stress and morphological remodeling in a hamster model [73]. In addition, CAF is more effective than HFD in causing PVAT-induced vascular dysfunction, associated with a significant reduction of vasodilatory response to acetylcholine in both mice [71] and rat [72]. Therefore, diet consisting of high fat, especially trans-fat and saturated fat can cause endothelial dysfunction and significantly increase the risk of cardiovascular diseases.

### 4.2. High-Sugar Diet

There is consensus that overconsumption of added sugar foods is positively associated with the risk for obesity and cardiovascular diseases [74, 75]. A prospective study has shown that people who got 17% to 21% of their calories from added sugar had a 38% higher risk of dying from cardiovascular disease compared with those who consumed 8% of their calories as added sugar [74]. Indeed, sugar added to foods and drinks supplies considerable extra calories without any benefits and may compromise the attainment of adequate dietary vitamin and mineral intake from the diet.

The new paradigm views overconsumption of sugar as an independent risk factor in cardiovascular diseases and other metabolic diseases [75]. High sugar intake may also cause endothelial dysfunction. Indeed, high glucose level promotes ROS formation, oxidative stress, and cellular death [76, 77]. Acute hyperglycemia caused by high glucose ingestion acutely deteriorates endothelial function in both human and animal studies, attributed by hyperglycemia-induced oxidative stress [78–81]. Exposure to high glucose increases eNOS activity and causing ROS formation due to eNOS uncoupling and aldose reductase activation in endothelial cells [82]. On the other hand, a recent double-blind randomized crossover trial has demonstrated that fructose load in healthy young subjects leads to unfavorable modifications of metabolic parameters, increased systolic blood pressure, and decreased endothelial NO production comparing to the same amount of glucose [83].

Another possible mechanism of how high-sugar diet induces endothelial dysfunction is attributed by advanced glycation end products (AGEs), a group of modified proteins and/or lipids that become glycated after exposure to sugars. AGEs can be ingested with high-temperature processed foods and also endogenously formed by nonenzymatic glycoxidation as a consequence of a high dietary sugar intake [84]. Chronic hyperglycemia promotes the formation of AGEs [85, 86], which have significant proinflammatory and prooxidant effects that contribute towards the pathology of diabetic and aging-related complications [87, 88]. In diabetic patients, high-AGE diet causes a significant increase in serum inflammatory markers [CRP and tumor necrosis factor *α* (TNF-*α*)] and endothelial dysfunction marker VCAM-1, whereas a low-AGE diet leads to a suppression of all these markers [89].

Another interesting mechanism by which high-sugar diet could promote oxidative stress is via protein phosphatase 2A (PP2A), a serine/threonine phosphatase that is responsible for the dephosphorylation of a wide range of substrates involved in cellular signaling, including p66Shc. High level of sugar has been shown to activate PP2A and NF-*κ*B [90, 91]. P66shc is a longevity protein that regulates ROS and apoptosis, while dephosphorylated p66Shc may translocate into mitochondria and trigger oxidative stress [92]. Conversely, inhibition of high glucose-mediated PP2A expression prevents oxidative stress and increases NO production, thus reducing endothelial dysfunction [90].

### 4.3. Ketogenic Diet/Low-Carbohydrate, High-Fat Diet

The ketogenic diet is a low-carbohydrate, high-fat and adequate protein diet described a few decades ago for the management of children with epilepsy [93]. A classic ketogenic diet consists of a ratio of 4 : 1 fat to carbohydrate and protein [94]. In recent years, ketogenic diet or low-carbohydrate, high-fat diet (LCHFD) is suggested to be a successful weight-loss tool for obese subjects [95, 96] and popular among healthy people to maintain bodyweight. The rationale behind this diet is to induce ketosis, which the shift to fatty acids as the main respiratory substrate leads to increased production of ketone bodies (acetone, acetoacetate, and *β*-hydroxybutyrate) [97]. Currently, there are some evidence showing that ketogenic diet possesses anti-inflammatory effects and leads to short-term improvements in some cardiovascular risk factors and reduction in blood pressure [98–100]. However, there are still lacks of promising results to show that ketogenic diet or LCHFD is beneficial to vascular function.

In endothelial cells, ketone bodies significantly induce the expression of genes involved in the cellular antioxidant defense system (Nrf2 and HO-1) and reduce DNA damage against oxidative insult [101]. Ketogenic diet can also reduce glucose metabolism and improve heart function in a mice model with endothelial-specific Notch inhibition. [102]. However, the beneficial effect of ketogenic diet or LCHFD is still questionable, especially in human studies. Indeed, in ApoE knockout mice, LCHFD has no effect on oxidative stress markers, but accelerates atherosclerosis and reduces endothelial progenitor cells [103]. A study of Swedish women suggests that low-carbohydrate diets are associated with an increased risk of cardiovascular diseases [104]. A case-control study shows that ketogenic diet can promote arterial stiffening and endothelial damage in children and young adults with epilepsy [105]. In addition, LCHFD shows no improvement of endothelial function (flow-mediated dilation) in normal weight, young, healthy women [106], while LCHFD may lead to a reduction in flow-mediated dilation and predispose the endothelium to hyperglycemia-induced damage in healthy young adults [107]. Indeed, the detrimental effect of ketogenic diet may be attributed to the formation of advanced glycation end (AGE) products, which promote vascular damages [108]. Although the beneficial effects of ketogenic diet or LCHFD on metabolic parameters are relatively promising, further researches are warranted to investigate the effect of ketogenic diet or LCHFD on cardiovascular health.

### 4.4. Mediterranean Diet

Mediterranean diet is characterized by high plant proteins, monounsaturated fat, and low animal products and saturated fat. The Mediterranean diet refers to an eating pattern of the olive growing areas surrounding the Mediterranean Sea. Numerous epidemiologic and intervention studies have shown that Mediterranean diet characterized by high consumption of vegetables, fish, olive oil, and moderate wine consumption is associated with a positive effect on endothelial function and a lower incidence of cardiovascular diseases [109–111]. Close adherence to a Mediterranean diet improves endothelial function (increased flow-mediated dilation) in obese subjects [109].

Indeed, Mediterranean diet is encompassed of nutrition and cultural behavior such as lifestyle and physical exercise [112]. The exact mechanism of which the Mediterranean diet has cardioprotective effects is thereby uncertain, but it is suggested that antioxidant and anti-inflammatory effect, improvement in endothelial function, and lipid profile are possible mechanisms [111]. Mediterranean diet has been shown to reduce blood pressure in hypertensive women and associate with increased plasma levels of NO and ET-1 and upregulated eNOS and ET-1 receptor in the endothelium [113]. Also, consumption of Mediterranean diet induces a reduction in endothelial damage and dysfunction, which is associated with an improvement in the regenerative capacity of the endothelium in healthy elderly subjects [114]. In randomized clinical trials, Mediterranean diet has been shown to significantly reduce circulating ox-LDL and inflammation markers in high cardiovascular risk subjects [115], as well as reduce oxidative stress and endothelial senescence in elderly subjects [116]. However, contradictory result has also been shown about the effect of the Mediterranean diet on endothelial function. In a clinical trial, treatment for 4 weeks with a Mediterranean-inspired diet fails to show beneficial effect in vascular function and reduce oxidative stress in healthy individuals with a low-risk profile for cardiovascular disease [117].

One special feature of Mediterranean diet is the consumption of olive oil, which is characterized by the high ratio monounsaturated to saturated fat. Consumption of olive oil-rich diet can decrease blood pressure and improve endothelial function in young women, which is associated with reduction in oxidative stress and inflammation mediators, such as ox-LDL and CRP levels [118]. However, our recent study suggests that olive oil, when compared to red fruit (*Pandanus conoideus Lam*) oil, has no significant effects on eNOS phosphorylation, NO production, and ROS levels in endothelial cells [119]. The favorable effect of Mediterranean diet on endothelial function might also be attributed to other components of this diet such as red wine and fish oil, as well as exercise which will be discussed below. Nevertheless, Mediterranean diet may represent a therapeutic strategy to reduce oxidative stress and inflammation and improve the associated metabolic and cardiovascular risk.

### 4.5. Calorie Restriction

Calorie restriction is the most potent and reproducible dietary interventions that shows beneficial effect in extending lifespan and attenuating aging-related chronic diseases including obesity and endothelial dysfunction [120].

In animal studies, caloric restriction is achieved by reducing calorie intake by 20-50% of *ad libitum* intake without altering the proportion of nutrients and inducing malnutrition [120]. Calorie restriction has been shown to improve life span, as well as physiological functions in most experimental models from yeast to nonhuman primates [121–123]. Short-term caloric restriction can prevent aging-induced endothelial dysfunction, at least partially by reversing altered iNOS/eNOS ratio, reducing oxidative stress, and increasing SOD enzyme activity in rat [124]. In both young and old mice, caloric restriction can reverse endothelial dysfunction by enhancing eNOS activity and NO production [125–127].

In human studies, lifelong caloric restriction has been shown to prolong lifespan, reduce atherosclerosis, and improve endothelial function [124, 128]. The most widely explored mechanisms of the beneficial effects of calorie restriction include improving cardiovascular risk-factor profile, reducing superoxide production and vascular oxidative stress, lowering circulating inflammatory cytokines, and upregulating sirtuin 1 (SIRT1) expression [129]. SIRT1 is a known longevity protein which can stimulate eNOS expression and activity [126, 130]. In response to caloric restriction, SIRT1 is activated and upregulates the activity of eNOS via deacetylating eNOS on lysine 496 and 506 residues [126, 131]. On the other hand, inhibition of SIRT1 prevents endothelium-dependent vasodilation and reduces NO bioavailability, suggesting that NO-mediating effect of caloric restriction is regulated via SIRT1 [132].

Although the translatability of caloric restriction to humans can be debatable, randomized trial has shown that a two-year 25% caloric restriction can attenuate biological aging and reduce the risk of cardiovascular diseases in nonobese young and middle-aged adults [133, 134]. Low-calorie diet can also improve endothelium-dependent vasodilation in obese patients with existing hypertension [135]. Long-term (>6.5 years) of caloric restriction has significantly reduced blood pressure comparing to that prior to the initiation [136]. Alternate day fasting with a low-fat diet for 12 weeks can effectively reduce weight and improve endothelial function (flow-mediated dilation) in both normal weight and overweight adults [137]. Collectively, these data suggest that short to long-term caloric restriction or reduced calorie intake may be able to prevent as well as reverse endothelial dysfunction and cardiovascular complications that caused by aging and obesity.

### 4.6. Diet Components and Supplements

#### 4.6.1. Alcohol and Wine

A large number of clinical trials and epigenetics studies have strongly correlated the long-term consumption of polyphenol-rich diet with protection against chronic diseases such as cancers and cardiovascular diseases [138–140]. Red wine contains numerous amounts of polyphenols which possess antioxidant and anti-inflammatory properties [141]. Resveratrol (3,5,4′-trihydroxy-trans-stilbene) is a plant polyphenol found mainly in grape fruits and red wine [142, 143]. Resveratrol is well-studied for its beneficial effect in cardiovascular protection by increasing NO production in the endothelium. Resveratrol can upregulate eNOS expression, stimulate eNOS activity, and prevent the uncoupling of eNOS [144]. In addition, resveratrol can improve endothelial function by activating SIRT1 [145]. Also, resveratrol has been shown to attenuate ox-LDL-induced cytotoxicity, apoptosis, ROS generation, and intracellular calcium accumulation in endothelial cells [39]. Therefore, red wine has been shown to improve endothelial function and reduce oxidative stress and the risk of cardiovascular events in both human and animal studies [146–148]. The beneficial effects of long-term consumption of moderate amounts of red wine can be attributed to increased HDL and reduction of ET-1 expression [149], as well as upregulation of sGC [150] and antioxidant enzymes (SOD and HO-1) [148]. Nevertheless, human clinical trials have shown that the beneficial effects of polyphenol are not always achievable within the context of moderate alcohol consumption as the bioavailability of the polyphenolic metabolites that reach the human body are always very low.

Indeed, recent studies suggest the potential influence of ethanol on endothelial functions [151, 152]. Both cross-sectional and randomized clinical trials have suggested that moderate alcohol consumption can improve endothelial function in healthy subjects [151, 153–155]. Chronic low ethanol consumption has also been shown to reduce systolic blood pressure and improve endothelial function in rat [156]. Moderate alcohol exposure (up to 1 drink or 12.5 g alcohol per day for women and 2 drinks or 25 g alcohol per day for men [157]) increases the activity of eNOS and NO production from the endothelium *in vitro* [158–160]. These studies suggest that ethanol may increase eNOS gene and protein expressions in endothelial cells. However, there are still controversies in the finding from the human studies. Flow-mediated dilation is impaired in either moderate or excessive alcohol consumption group according to a Japanese study [161]. Although the results from clinical studies of moderate/habitual alcohol consumption remain controversial, one must bear in mind that excessive alcohol consumption is unquestionably harmful to human health at several levels.

#### 4.6.2. Coffee

Coffee is one of the most popular pharmacologically active beverages, which is rich in plant phenolic compounds [162]. Various epidemiologic studies have already shown inverse associations of regular consumption of coffee with metabolic diseases including obesity and type 2 diabetes, as well as with cardiovascular diseases [163–166]. The beneficial effect of coffee in improving vascular function appears to be attributed by reducing the ROS production and enhancing NO bioavailability [167]. In a randomized, placebo-controlled, cross-over study, flow-mediated dilation response is significantly improved in the subjects taking caffeinated coffee compared to both groups taking decaffeinated coffee or water, suggesting that the consumption of caffeinated coffee can improve endothelial function [168]. Moreover, the acute administration of caffeine augments endothelium-dependent vasodilation in healthy young men associated with an increased NO production [169]. Indeed, coffee has been described as probably the most relevant source of dietary antioxidants [170]. A few studies have documented that a single serving of coffee may increase plasma antioxidant capacity by around 5% [170–172]. Caffeine may enhance endothelial cell migration and reendothelialization in part through an AMPK-dependent mechanism [173]. The most abundant polyphenol in coffee, chlorogenic acid (CGA), has been shown to increase the production of NO and reduce oxidative damages in isolated mice aortic ring [174]. However, the effects of coffee consumption on endothelial function are still controversial. Some studies have suggested an adverse effect of coffee consumption in endothelial function and blood pressure [172, 175]. These controversies may be attributed by the different additional supplements added to the coffee, including sugar and milk. Nevertheless, as coffee is a popular beverage worldwide, the effect of caffeine on endothelial function warrants detailed researches.

#### 4.6.3. Gluten

Gluten consists of two classes of proteins, prolamin, and glutenin and can be found in several kinds of cereal. Gliadin, the wheat prolamin, is not fully digested by intestinal enzymes and is metabolized to biologically active peptides with cytotoxic activities, including increased intestinal permeability and modulation of the immune system [176]. Celiac disease is an autoimmune and systemic disease that develops in genetically susceptible individuals as a result of a permanent intolerance to gluten [177]. Celiac disease is also associated with endothelial dysfunction [178]. Gluten-free diet can reduce the risk for endothelial dysfunction [179] and reduce oxidative stress [180] in patients with celiac disease.

In individuals without celiac disease, the physiological effect of gluten intake is relatively unknown. Wheat alpha-amylase/trypsin inhibitors (ATIs) are activators of the innate immune system via activating toll-like receptor 4 (TLR4) in myeloid cells, triggering several autoimmune/inflammatory diseases. Gluten-containing cereals have been shown to contain the high concentration of ATIs that activate TLR4 [181]. Wheat ATIs are implicated in the pathogenesis of celiac disease as well as nonceliac wheat sensitivity [182]. Mice on a gluten-free diet show significantly attenuated clinical parameters of kidney dysfunction (proteinuria, haematuria, and hemoglobinuria) and serum inflammatory cytokines (IL-6 and TNF-*α*) compared to mice on a gluten-containing diet [183]. Increased gluten intake is associated with increased concentrations of plasma inflammatory marker, *α*_2_-macroglobulin, in young adults without celiac disease [184]. Also, gluten diet can exacerbate vascular oxidative stress and inflammation and accelerate atherosclerosis in ApoE knockout mice [185]. Gluten feeding has been shown to elevate the rate of superoxide and nitrotyrosine production in the aortic root lesion [185]. These results suggest that the potential use of gluten-free diet is an alternative to ameliorate cardiovascular diseases independent of celiac disease.

#### 4.6.4. Dark Chocolate

The main compound in chocolate is cocoa, which contains high content of polyphenols, while dark chocolate contains a greater amount of cocoa compared with milk chocolate [186]. Chocolate and cocoa have been described as one of the most important sources of flavonoids in the human diet [187, 188]. Data currently available suggests that daily consumption of cocoa-rich chocolate may partially reduce the risk of cardiovascular disease [189].

Several mechanisms of how chocolates or cocoa flavonoids are protective against cardiovascular disease have been proposed including improving endothelial function, decreasing blood pressure, possessing antioxidant, antiplatelet, and anti-inflammatory effects, as well as a positive modulation of insulin resistance [187, 190–192].

Cocoa has been shown to reduce oxidative stress via lowering the activation of NADPH oxidase and stimulating NO-mediated vasorelaxation [193, 194]. In a cross-sectional study, administration of dark chocolate (>85% cocoa), but not milk chocolate (<35% cocoa), can significantly improve flow-mediated dilation and NO bioavailability in patients with nonalcoholic steatohepatitis [195]. In another study, dark chocolate, but not milk chocolate, inhibits platelet activity and oxidative stress in smokers [194]. And both studies suggest that dark chocolate reduces oxidative stress by downregulating NOX2.

Administration of a single dose of cocoa is dose dependently associated with significant increases in circulating flavanols and flow-mediated dilation in medicated type 2 diabetes patients [190]. In a randomized trial, the acute ingestion of either solid dark chocolate or liquid cocoa improves endothelial function and reduces blood pressure in overweight adults, while sugared cocoa may have attenuated these beneficial effects [196]. These studies suggest that the consumption of sugar-free dark chocolate would be the ideal choice to prevent endothelial dysfunction and cardiovascular diseases.

#### 4.6.5. Omega 3 Polyunsaturated Fatty Acids

The correlation between high omega 3 polyunsaturated fatty acids (n-3 PUFAs) and low incidence of cardiovascular disease has been long recognized since an epidemiological study in the 1970s [197]. The broad range of beneficial effects of n-3 PUFAs is well known, including antiatherogenic, antithrombogenic, and blood pressure lowering [198]. Replacement of saturated fat with unsaturated fat has been suggested to improve endothelial function and show beneficial effect on endothelial repair and maintenance [199]. One of the mechanisms by which n-3 PUFAs influence endothelial function is mediated by their incorporation into biological membrane phospholipids and modulation of membrane composition and fluidity [200]. Caveolae-associated receptors mediate important pathways such as the NO-cGMP pathway, and the NADPH oxidase and TNF-*α*-NF-*κ*B mediated COX-2 and PGE_2_ activation pathway [201, 202]. n-3 PUFAs may exert their beneficial effects in the endothelium, by modulating the composition of membrane caveolae, similar as other lipids [203].

n-3 PUFAs including eicosapentaenoic acid (EPA) and docosahexaenoic acid (DHA) have been shown to protect the cardiovascular system, in part, by enhancing eNOS activity and NO production [204, 205]. In various animal models, n-3 PUFAs have been shown to increase NO production by directly stimulating eNOS gene and protein expression, which improve vasodilation [206–208]. In addition to enhancing NO production, n-3 PUFAs also decrease oxidative stress. In endothelial cells, n-3 PUFAs can reduce the levels of inflammatory cytokines (IL-6 and TNF-*α*) and oxidative stress markers (ROS and malondialdehyde (MDA)) and increase the activity of SOD [209]. EPA and DHA have been shown to attenuate oxidative stress-induced DNA damage in endothelial cells through the upregulation of NRF2-mediated antioxidant response [210]. In epidemiology studies and clinical trials, n-3 PUFA supplementation prevents the development of atherosclerotic diseases [211, 212]. Moreover, n-3 PUFA supplementation has been shown to improve endothelial function in patients with primary antiphospholipid syndrome [213], type 2 diabetes [214], peripheral arterial disease [215], heart failure [216], and hypertriglyceridemia [217].

Recently, a ratio of EPA : DHA 6 : 1 has been recognized as a potent formulation to improve endothelial functions in different studies. Intake of omega-3 formulation EPA : DHA 6 : 1 can restore endothelium-dependent NO-mediated relaxations, most likely, by preventing the upregulation of the local angiotensin system, NADPH oxidase, and the subsequent formation of ROS in old rats [218]. Also, EPA : DHA 6 : 1 can prevent endothelial senescence in middle-aged or old rats by limiting both the shedding of endothelial microvesicles and their prosenescent, prothrombotic, and proinflammatory effects in endothelium [218]. However, a recent clinical study suggests that high-dose n-3 PUFA treatment in very high-risk patients with atherosclerotic cardiovascular disease and type 2 diabetes cannot improve the endothelial function indices [219]. Therefore, stronger evidence is needed before large-scale prescription of n-3 PUFAs in very high-risk patients. In overall, n-3 PUFAs have high potential to beneficially impact endothelial function and cardiovascular outcome.

#### 4.6.6. Vitamins

Antioxidant vitamins (vitamins C, vitamin E, and *β*-carotene) appear to have beneficial effects on vascular endothelial function [220, 221]. *β*-carotene belongs to the family of carotenoids which are lipid-soluble plant pigments. *β*-carotene has been demonstrated to reduce inflammatory response and oxidative stress in TNF-*α*-treated endothelial cells *in vitro* [222], to prevent LDL oxidation [223], and to reduce the risk of atherosclerosis [224] and the incidence of cardiovascular diseases [223, 225, 226]. It has been shown that vitamin C (a water soluble antioxidant) and vitamin E (a lipid-soluble antioxidant) improve endothelial function by normalizing the expression of eNOS in hypercholesterolemic pigs [227]. Simultaneous administration of vitamins C and E can prevent the deleterious effects of postprandial hypertriglyceridemia on endothelial-dependent vasodilation [77].

In a randomized, placebo-controlled study, supplementation containing vitamin C (1000 mg/d) and vitamin E (400 IU/d) shows beneficial effects on glucose and lipid metabolism, blood pressure, and arterial elasticity in patients with cardiovascular risk factors [228]. Possible mechanisms for the beneficial effects of vitamins C and E on endothelial function could be attributed by eliminating superoxide and scavenging lipid hydroperoxyl radicals, and thereby maintaining NO bioavailability [220]. Long-term consumption of vitamin C in diabetic patients can improve certain echocardiographic parameters and enhance vascular endothelial function (flow-mediated dilation) [229]. Chronic consumption of vitamin E partially prevents hyperglycemia-induced endothelial dysfunction in diabetic rat, while vitamin E deficiency enhances diabetic endothelial dysfunction dramatically *in vivo* [230].

Combined treatment with vitamins C and E has beneficial effects on endothelium-dependent vasodilation and arterial stiffness, which are associated with changes in plasma markers of oxidative stress in essential hypertensive patients [231]. However, one should be cautious about the long term or high-dose usage of vitamin. Vitamin C has been shown to increase the production of AGE products in diabetic patients [232]. Long-term treatment with 1,800 IU of vitamin E has no beneficial effects on endothelial or left ventricular function in diabetic patients, and some vitamin E-treated patients has a worsening in some vascular reactivity measurements when compared with control subjects [231].

## 5. Physical Exercise

Sedentary lifestyle and overconsumption of energy-rich food have been identified as a risk factor for the development of some cardiovascular complications [233, 234]. In comparison, regular physical activity (child and adolescents: 60 mins of moderate-to-vigorous physical activity daily; adults: 150 to 300 mins of moderate intensity per week [235]) has long been considered necessary for the achievement and maintenance of optimal health. Prospective studies provide direct evidence that a physically active lifestyle delays all-cause mortality, extends longevity [236], and reduces risk for cardiovascular mortality by 42–44%, compared to sedentary lifestyle [237]. The terms “physical activity” and “physical exercise” refer to body movements by the skeletal musculature and associated with the consumption of energy. Specifically, the term “physical training” indicates a regular, structured physical activity to improve and/or maintain physical fitness and well-being [238]. Exercise training elicits beneficial effects in a number of physiological adaptations, including maximal oxygen consumption (VO_2max_), cardiac output, and maximal oxygen extraction, as well as maximal skeletal muscle blood flow capacity [239]. It is commonly recognized that physical training has beneficial effects on body composition and health, especially for weight loss [240], and also known as a nonpharmacological treatment of cardiovascular diseases [241].

### 5.1. Physical Exercise and Laminar Shear Stress

Regular exercises result in numerous health benefits, such as improving body composition and endothelial function and preventing insulin resistance, oxidative stress, and arterial hypertension [241]. One of the important mechanisms to improve endothelial function during exercising is the increased blood flow and shear stress which can improve vascular homeostasis by reducing the production of ROS and increasing the bioavailability of NO in the endothelium (discussed above) [242]. During exercising, repeated episodes of increased blood flow elicit an improvement in endothelial function and lead to the long-term benefits of regular exercise that prevent risk of cardiovascular diseases [243]. This mechanism is likely to involve chronic upregulation of NO production mediated by an enhanced expression of eNOS.

It is suggested that shear stress-mediated effects and consequent production and bioactivity of NO differ qualitatively and quantitatively according to the exercise involved [244]. Vascular laminar shear stress increases during exercise and is associated with a rapid upregulation of eNOS gene and protein expressions [245]. Varied durations of exercise training seem to influence the response of arteries to increase flow and shear stress [244]. It has been reported that improvement in NO-related vasodilation is observed in short- to medium-term training, whereas longer-term training is associated with arterial remodeling [244]. It is also important to note that laminar shear stress due to exercise has a predominant antioxidant effect and improves endothelial function, while oscillatory shear stress in hypertension is associated with opposite effects promoting oxidative stress and oxidative vasculature damage through a progressive increase in NADPH activity [246].

### 5.2. Physical Exercise, Arterial Pressure, and Oxidative Stress

Indeed, exercise is a double-edged sword for endothelial function [247]. Blood pressure distends arteries and causes stretching in vascular cells. Changes in pressure consequently generate circumferential stress (strain) in the compliant arteries. During exercise, because of increased heart rate and systolic pressure, expansion of arteries can induce cyclic circumferential strain on endothelial cells [248, 249]. Cyclic circumferential strain can also be increased by the relaxation of vascular smooth muscle, which induces vasodilation and stretching of the endothelium. The effects of cyclic circumferential strain are complex and variable. Increased exposure to cyclic circumferential strain has been shown to alter gene expressions in the endothelium [including eNOS and EDHF synthase (CYP450)] and promote the production of ROS, the expression of ICAM, selectin, and monocyte chemoattractant protein-1 (MCP-1) [250]. Indeed, chronic high blood pressure is associated with endothelial dysfunction and progression of atherosclerosis [251]. The major effect of increased cyclic circumferential strain on endothelial cells appears to be ROS-mediated proatherogenic [252]. The pattern of change in cyclic circumferential strain is relevant as transient increases in blood pressure and ROS production, which is associated with exercise bouts and may increase eNOS expression and other beneficial effects of exercise, whereas chronic increases in blood pressure may chronically elevate ROS, causing maladaptation [249]. Although it has been suggested that short-term exercise can cause oxidative stress by increasing LDL susceptibility to oxidation or vascular superoxide production [253], long-term and/or regular exercise has been shown to reduce oxidative stress and upregulate the expression of SOD [254] and other antioxidant defenses in human, which leads to positive arterial remodeling to normalize blood pressure and shear stress [255–258].

### 5.3. Physical Exercise and Arterial Remodeling

Arterial remodeling is the active process of structural alteration that occurs as a result of cell death, proliferation, and migration as well as changes in the extracellular matrix of a vessel and is controlled by the crosstalk between endothelium and vascular smooth muscle cells [259]. Sensitivity to shear stress of endothelium is important in arterial remodeling and can activate signaling pathways in vascular smooth muscle cells [259]. Vasculature in hypertensive individuals undergoes accelerated vascular wall thickening and leads to degeneration and calcification of the vascular wall and vessel stiffening, while compensatory vessel wall enlargement is observed in atherosclerotic patients [260].

**Figure 2:**
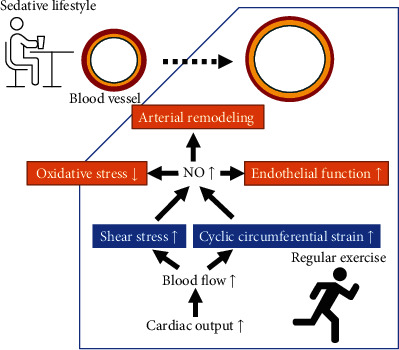
Regular exercise leads to arterial remodeling that contributes to cardiovascular performance and health benefits. During exercise, cardiac output and blood flow are increased, which generate shear stress and cyclic circumferential strain on arterial wall. Long period of exercise results in a long-term upregulation of eNOS. NO-mediated arterial remodeling results a chronic increase in vessel caliber, which structurally normalizes shear stress and cyclic circumferential strain

NO is an important endothelial regulator of flow- and pressure-induced arterial remodeling [260], while the congenital absence of eNOS causes adverse vascular remodeling [24, 261]. Upon long-term exercise training, NO-mediated structural adaptation occurs in the arteries, resulting in a chronic increase in vessel caliber, which structurally normalizes shear stress and cyclic circumferential strain (Figure 2). NO function is then returned towards baseline levels [262]. This process constitutes a long term and structural change mechanism for reducing shear stress, allowing NO bioactivity to return towards pretraining levels [244]. Moreover, positive arterial remodeling serves to enhance muscle performance by increasing the oxygen-exchange capacity and by increasing blood flow capacity. These adaptations potentially contribute to cardiovascular performance and health benefits in individuals who have long-term exercise training [262].

### 5.4. Physical Exercise in Animal Models

In healthy rats, a single resisted exercise session can improve endothelial function and NO synthesis in both the endothelium and the smooth muscle layer of mesenteric and promote insulin-induced vasodilation [263]. It is proposed that exercise stimulates factors that increase endothelial NO production including vascular distension, catecholamine release, and intermittent hypoxia. Exercise training in pigs [264] and hypertensive rats [265] has been shown to increase both gene and protein expression of eNOS. It is also suggested that the stimulation of NO production is dependent on the volume of exercise, and a greater demand of oxygen and nutrients is involved in the beneficial effects of exercise on the endothelium [263, 266]. Moreover, similar results are observed in hypertensive rats that single session of resisted exercise activates eNOS and promotes vasorelaxation [267]. Interestingly, expression of the prooxidant enzyme, NADPH oxidase, is reduced by exercise training in hypertensive rats, which may have a beneficial effect on the half-life of NO in the vascular wall [266]. These data reinforce that exercise can improve endothelial function, probably by stimulating NO production, even in hypertension. In diabetic rats, exercise training has been shown to normalize the diabetes-related endothelial dysfunction and improve insulin sensitivity [268, 269]. Moderate-intensity exercise reverses diabetes-related endothelial dysfunction independently of improvements in body weight or hyperglycemia in db/db mice. It is suggested that upregulations of eNOS and cytosolic Cu/Zn-SOD, but not MnSOD, play important roles in improving NO bioavailability, as well as in reversing endothelial dysfunction in diabetes via exercise [270]. In aging rats, exercising training increases sensitivity of blood vessel to shear stress and promotes acetylcholine-induced endothelial-dependent vasorelaxation [271], suggesting that exercising training is able to reverse age-related decline in vascular function.

In addition, long period of exercise can reduce the contractile response of the aorta to noradrenaline and increase the relaxation induced by acetylcholine in healthy rats [272–274], suggesting that the time of exercise exposure may also be critical to determine the beneficial effects of exercise on endothelial function. Chronic exercise training improves aortic endothelial and mitochondrial function through an adenosine monophosphate-activated protein kinase *α*2 (AMPK*α*2)-dependent manner in mice, which is also associated with increased mitochondrial antioxidative capacity (increased expression of MnSOD and catalase) [275]. These experimental studies suggest that physical exercise plays an important role in the prevention and treatment of endothelial dysfunction. However, additional studies are needed to establish the best kind, intensity, and duration of exercise to allow more efficient prescribing in clinical area.

### 5.5. Physical Exercise in Human Studies

In type 2 diabetic patients, lower-intensity exercise has physiological meaningful effects on endothelial function, while low to moderate intensity and aerobic exercises significantly increase flow-mediated vasodilation more than moderate- to high-intensity exercises and combined aerobic and resistance exercises, respectively [276]. Regular physical activity has been shown to promote the activities of antioxidant enzymes and stimulate glutathione levels in body fluids [277, 278]. In young prehypertensive patients, resistance training increases flow-mediated dilation and reduces blood pressure [279], as wells as improves resistance artery endothelial function and prooxidant/antioxidant balance [280]. In a recent randomized clinical trial, aerobic exercise training, resistance training, and combined training have also shown similar beneficial in improving endothelial function but impacts on ambulatory blood pressure appear to be variable in middle-aged and older individuals with prehypertension or hypertension [281].

Aerobic exercise increases both gene and protein expression of eNOS in patients with coronary artery disease [245]. Six-month exercise training reduces arterial blood pressure and is associated with increased NO content (determined by plasma nitrite/nitrate levels) in hypertensive women [282]. However, biological sample acquisition from humans subjected to physical exercise is challenging, thereby the changes in NO production are predominantly evaluated based on the measurements of NO content in the exhaled air. Physical training is associated with an increase in NO content in the exhaled air [283], while some studies suggest a reduced NO content in the exhaled air after physical exercise [284–286]. This controversy is unsurprising due to the complexity of NO exchange and multisystemic nature of the physiological responses to physical exercise [249].Nevertheless, variations of NO content in the exhaled air may also depend on the levels of physical activity [284].

### 5.6. High-Intensity Exercise: Good or Bad?

It is clinically important to select the appropriate intensity, duration, frequency, and kind of exercise, as high-intensity exercise can be harmful to human vessels [287]. In general, the guidelines for patients with mild-to-moderate essential hypertension recommend exercise at an intensity of around 50% of maximum oxygen consumption, such as walking, jogging, cycling, or swimming, for 30 minutes and 5 to 7 times per week [287–289]. In recent years, low-volume high-intensity exercise training has become advocated with data showing comparable benefits to traditional endurance-based training in skeletal muscle metabolic control and cardiovascular system function [290]. Aerobic training of high intensity, compared to the aerobic training of low intensity and controls, has been shown to improve endothelium function (flow-mediated vasodilatation) in patients with metabolic syndrome or diabetes [291]. Both continuous moderate-intensity aerobic exercise and high-intensity interval aerobic exercise can significantly improve endothelial function, in terms of flow-mediated dilation, the carotid femoral pulse wave velocity and the femoral dorsalis pedis pulse wave velocity in health men [292]. A recent randomized controlled, crossover study also suggests that short-duration maximal intensity exercise has comparable effects on endothelial function and oxidative stress with mild and moderate exercise [293].

**Figure 3:**
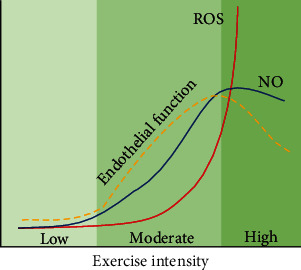
Schematic of the effect of different exercise intensities in vascular nitric oxide (NO) and reactive oxygen species (ROS) level and endothelial function. Low-intensity exercise may have minimal effect on NO and ROS production and physiological meaningful effects in endothelial function. During moderate-intensity exercise, production of NO is augmented while ROS production is increased in a slower rate, resulting in improvement of endothelial function. During high-intensity exercise, production of ROS is significantly greater that of NO, resulting in reduced endothelial function.

On the other hand, collective evidence has suggested that production of ROS is greater than production of NO during high-intensity exercise, resulting in reduced endothelial function (Figure 3). For example, high-intensity exercise training has no beneficial effects on endothelial function in spontaneous hypertensive rat, but rather augments oxidative stress, resulting in eNOS uncoupling and ROS production and leading to further decrease in NO bioavailability and increase in ROS [294]. High-intensity exercise also increases the indices of oxidative stress, including plasma concentration of 8-hydroxy-2′-deoxyguanosine (8-OHdG) and serum concentration of ox-LDL, and decreases endothelium-dependent vasodilation in healthy men [295]. It is suggested that the massive increase in oxygen uptake that occurs in skeletal muscle during high-intensity exercise is associated with an increase in ROS [296]. Moreover, oxidation of tetrahydrobiopterin (BH_4_) is suggested to be responsible in oxidative stress-induced eNOS uncoupling during high-intensity exercise. BH_4_ deficiency is associated with the ROS production from dysfunctional eNOS; superoxide produced by uncoupled eNOS which also inactivates NO [297]. It has also been reported that the degradation of BH_4_ by ROS is associated with the inhibition of eNOS [297, 298]. These findings suggest that BH_4_ deficiency-induced eNOS dysfunction causes endothelial dysfunction in hypertension through promoting oxidative stress. It is possible that high-intensity exercise activates oxidative stress through exacerbation of BH_4_ deficiency, as well as oxidation of BH_4_. These data may suggest that high-intensity exercise increases oxidative stress in humans, which diminishes endothelium-dependent vasodilation.

Recently, high-intensity interval training (HIIT) has been proposed to have positive effects on metabolic profile and improves cardiovascular health [299–301] and used as an alternative to traditional endurance training to promote metabolic and cardiovascular health. The detailed mechanisms responsible for the beneficial effect of HIIT are not well known, but one proposed mechanism is that HIIT increases aerobic capacity and thus delays the onset of exhaustion [302]. The common formula of HIIT involves a 2 : 1 work-to-recovery ratio [303]. Interval running (30 s at VO_2max_ alternated with 30 s at 50% VO_2max_) has been shown to provide a greater exercise training stimulus than continuous running to improve VO_2max_ [304]. In general, HIIT elicits greater aerobic capacity adaptations compared to chronic training in improving cardiovascular risk [303]. Recently, HIIT has been shown to improve arterial stiffness, cardiovascular health, and metabolic profiles in inactive individuals with obesity and type 2 diabetes [305–307]. Future studies are warranted to investigate the effects and underlying mechanisms of HITT on endothelial function in a different pathophysiological condition, including aging, diabetes, and hypertension.

### 5.7. Exercise and Diet Combination

In order to maximize the beneficial effects of exercise on vascular function, the combination with a healthy diet is reasonable. Most current studies support that exercise is effective to ameliorate HFD-induced endothelial dysfunction and improve microvascular reactivity in young, healthy men [308], as well as to improve insulin action and reductions in glycemia and prevent endothelial dysfunction after high-sugar-food ingestion with endurance exercise performed on the previous day [80]. In addition, it is also suggested that the combination of supervised diet and exercise training is effective to improve vascular function and multiple adolescent obesity-related end points [309]. In addition, interval exercise combined with a low-calorie diet improves endothelial function in obese adult female [310]. Moreover, a recent study has demonstrated that aerobic exercise prior to a high-sugar meal has no improvement on endothelial function, blood glucose, insulin, ET-1 or NO concentrations, or insulin sensitivity in postmenopausal women [311]. Therefore, it is synergistic to improve vascular function when proper exercise is carried out in parallel with a healthy diet.

## 6. Conclusions

Healthy lifestyle and diet are important in reducing the risk for metabolic and cardiovascular diseases. A large body of evidence underlines the importance of proper diet and physical exercise in preventing oxidative stress and endothelial dysfunction, which are risk factors for cardiovascular diseases. Diet is an important source for antioxidants. In general, consumption of balanced diet with reduced amount of added sugar and saturated fat has been shown to reduce oxidative stress and promote endothelial function. However, it has been suggested that the indiscriminate use of antioxidants may even be harmful, since basal levels of ROS are imperative for certain cellular functions [312]. Overconsumption of certain nutrients as well as overintensive exercise may impede some essential cellular defense mechanisms. A fine balance between oxidative stress and antioxidants is important for normal function in the cells, and interfering with this balance may lead to unfavorable effects. While the best diet composition and exercise intensity may vary among individuals and physiological conditions, further detailed studies are needed. Thereby, before the ultimate problem is solved, a balanced diet and regular exercise are always helpful.
